# Associations Between 24-h Movement Behaviors and Macronutrient Intake Among Students Aged 6–17 Years: Insights from the China Health and Nutrition Survey

**DOI:** 10.3390/nu17203262

**Published:** 2025-10-17

**Authors:** Zekai Chen, Lin Zhu, Ziqi Chen, Jialin Quan, Zhuofan Zhang

**Affiliations:** 1 School of Sport and Health, Guangzhou Sport University, Guangzhou 510500, China; zekaichen1993@163.com (Z.C.); chenziqipt@163.com (Z.C.); 15113855407@163.com (J.Q.); zhuofanzhang2001@163.com (Z.Z.); 2 Research Center for Innovative Development of Sports and Healthcare Integration, Guangzhou Sport University, Guangzhou 510500, China; 3 Innovative Research Center for Sports Science in the Guangdong-Hong Kong-Macao Greater Bay Area, Guangzhou Sport University, Guangzhou 510500, China

**Keywords:** 24-h movement, macronutrient, Dietary Reference Intakes, dose-response

## Abstract

**Background/Objectives:** This study aims to examine the relationships between 24-h movement guideline (24HMG) adherence and macronutrient intake, as well as assess dose–response relationships between 24-h movement behaviors and macronutrient intake among students aged 6–17 years. **Methods:** The study included 3624 participants aged 6 to 17 years from four rounds (2004–2011) of the Chinese Health and Nutrition Survey (CHNS). Participants’ 24-h movement behaviors and dietary intakes were evaluated. **Results:** Adherents to physical activity (PA) guideline had higher carbohydrate, fat, and protein intake (all *p* < 0.05). Those following the screen time (ST) guideline had a higher percentage of dietary energy intake (E%) from carbohydrates but a lower percentage from fat (all *p* < 0.05). Sleep (SLP) guideline adherents demonstrated lower protein intake and E% (all *p* < 0.05). PA guideline adherents were less likely to exceed carbohydrate Dietary Reference Intakes (DRIs) (OR = 0.83, 95% CI: 0.69–0.99), but more likely to surpass fat DRIs (OR = 1.20, 95% CI: 1.02–1.40). ST guideline adherents were more likely to exceed carbohydrate DRIs (OR = 1.32, 95% CI: 1.11–1.56) and less likely to surpass fat DRIs (OR = 0.78, 95% CI: 0.68–0.91). Dose–response analyses showed that moderate-to-vigorous physical activity (MVPA) and ST had positive linear associations with carbohydrate intake below DRIs. ST also showed positive linear associations with fat intake above DRIs. MVPA showed a nonlinear relationship with fat intake above DRIs. **Conclusions:** Among Chinese children and adolescents aged 6–17 years, those who meet the PA guideline should be cautious about the risk of excessive fat intake, while those adhering to the ST guideline should be aware of the risk of excessive carbohydrate intake in their daily diet. For promoting health and maintaining balanced macronutrient intake, MVPA should range from 60 to 90 min per day. This study underscores the importance of adjusting macronutrient intake according to levels of 24-h movement behaviors, especially MVPA and ST.

## 1. Introduction

The burden of chronic diseases among children and adolescents is increasing globally, with non-communicable diseases (NCDs) accounting for one-third of disability-adjusted life years (DALYs) in the total disease burden among populations under 20 years old worldwide [1,2]. Inadequate physical activity (PA), insufficient sleep (SLP), and sedentary behaviors are considered to be closely linked to NCDs [3,4,5]. In recent years, the relationship between adherence to the 24-h movement guidelines (24HMG) and health outcomes in children and adolescents has gained global attention [6,7,8]. The 24HMG include behavioral indicators such as physical activity (moderate-to-vigorous physical activity, MVPA), screen time (ST), and SLP. Consistent research shows that meeting these guidelines is significantly associated with various positive health outcomes among Chinese children and adolescents [9,10,11]. Despite these advantages, current data indicate that less than 10% of children and adolescents in China meet all three components of the 24HMG [12].

Macronutrients (carbohydrates, protein, and fats) are essential for the growth and disease prevention of children and adolescents [13,14,15]. Research shows that maintaining a balanced intake of these macronutrients during childhood and adolescence can promote healthy weight management habits and lower cardiometabolic risks [16,17,18]. Conversely, improper macronutrient intake, such as excessive fat consumption, is linked to adverse health outcomes, including obesity and insulin resistance [19,20]. Notably, since the late 20th century, Chinese children and adolescents have exhibited a trend of consuming excessive fat and insufficient protein and carbohydrates in their diet [21].

Dietary intake among children and adolescents is closely linked to movement behaviors. Evidence indicates that ST is associated with unhealthy snacking [22], while lack of PA and inadequate SLP are related to increased energy intake [23]. In 2024, a study from Spain found that children who met all three 24HMG criteria showed higher adherence to Mediterranean dietary patterns [24]. Furthermore, research suggests a dose–response association between the number of recommendations met and fruit and vegetable intake among adolescents [25]. These prior study results indicate that the relationship between 24-h movement behaviors and dietary intake in children and adolescents might be intricated and remains inadequately explored. Although the 24HMG and Dietary Reference Intakes (DRIs) provide independent recommendations for behaviors and dietary intake, respectively, there are still some research gaps, such as whether children and adolescents who meet the movement guidelines are more likely to follow the macronutrient recommendations in Dietary Reference Intakes, whether children and adolescents with different levels of adherence to the 24-h movement guidelines show significant differences in macronutrient intake, and whether there is a dose–response relationship between 24-h movement behaviors and macronutrient intake, etc. These research gaps hinder the development of comprehensive strategies that integrate movement behaviors and dietary behaviors to enhance the health of children and adolescents. Understanding the relationship between 24-h movement behaviors and macronutrient intake will offer an essential theoretical basis for future health promotion efforts. As a result, this evidence could better address the current global trend of rising chronic diseases, such as obesity, hypertension, and hyperlipidemia, which are increasingly affecting younger populations.

Therefore, this study aims to investigate the association between adherence to the 24-h movement guidelines and macronutrient intake, and to assess dose–response associations between these movement behaviors and macronutrient intake among students aged 6–17 years. The findings will provide scientific evidence for developing comprehensive intervention strategies to promote healthier lifestyles among children and adolescents.

## 2. Materials and Methods

### 2.1. Study Design and Participants

**Figure 1:**
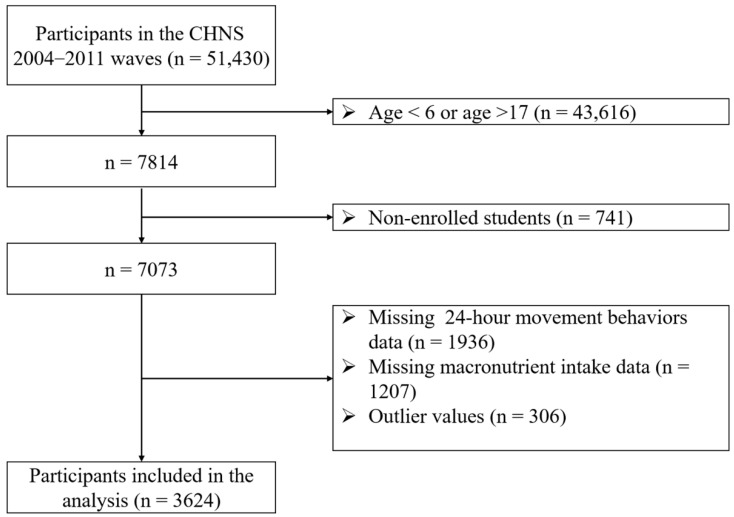
Flow chart of this study.

This study uses data from the China Health and Nutrition Survey (CHNS), an ongoing open cohort designed to examine the impact of health, nutrition, and family planning policies implemented by both national and local governments. The CHNS has been conducted since 1989, with follow-ups approximately every 2 to 3 years, the most recent occurring in 2015. A multistage, random cluster sampling method was used to select around 7200 households, including over 30,000 individuals across 15 provinces and municipalities. Additional details about the CHNS are available on the official website (https://chns.cpc.unc.edu/about/design/, accessed on 15 August 2025) and in previously published studies [26]. Since sedentary behavior data in the CHNS were first collected in 2004, and publicly available macronutrient data extend only through 2011, considering the availability of samples in the database and ensuring that participants have complete 24-h movement behavior data, this study analyzed four survey waves covering the period from 2004 to 2011. The survey received approval from the institutional review boards at both the University of North Carolina at Chapel Hill and the National Institute for Nutrition and Health (NIHH) under the China Center for Disease Control and Prevention (CCDC) [27]. All parents provided written informed consent for their children’s participation. Figure 1 presents the flow chart.

### 2.2. Assessment of Macronutrient Intake

In the CHNS, participants’ intake of macronutrients, including carbohydrates, fats, and proteins, was evaluated through three consecutive 24-h dietary recalls [28]. Trained health workers conducted in-person interviews with participants, using food models and visual aids to record the type of food, amount, meal type, and place of consumption for all items eaten in the previous 24 h. Participants aged 12 years and older were asked to recall the foods they had consumed during the last 24 h. For those under 12, a mother or maternal guardian responsible for food preparation and feeding in the household reported the children’s food intake [29]. In the CHNS, the total energy intake from 24-h dietary recalls was validated using the doubly labeled water method [30].

The average intakes of dietary energy (kcal/day), carbohydrates (g/day), fats (g/day), and proteins (g/day) were calculated using the Chinese Food Composition Table (CFCT) [31]. The calculations for the percentage of dietary energy intake (E%) are as follows: carbohydrate E% = ([carbohydrate (g) × 4]/total energy intake [kcal] × 100), protein E% = ([protein (g) × 4]/total energy intake [kcal] × 100), and fat E% = ([fat (g) × 9]/total energy intake [kcal] × 100). According to the recommended Dietary Reference Intakes, the energy contribution ratios for carbohydrates, fats, and proteins in the daily diet of Chinese children and adolescents aged 6 to 17 years should be 50% to 65%, 20% to 30%, and 10% to 20%, respectively [32]. Based on these standards, the intake levels of each macronutrient for the participants were categorized as below, meeting, or above the recommended values.

### 2.3. Assessment of 24-h Movement Behaviors

The behavior assessment questionnaire used in CHNS was developed by adapting the US Health Interview Surveys, with input from Professor Barry Popkin of the University of North Carolina and Professor Barbara Ainsworth of Arizona State University [9]. The assessment of physical activity includes several categories: in-school physical activity, before- and after-school physical activity, commuting, and household chores. Physical activities mainly include various ball sports (e.g., basketball, soccer, badminton), gymnastics, dance, running, and other common forms of exercise. Commuting activities involve walking and cycling, while household activities include cooking, laundry, and house cleaning. Participants’ physical activities during weekdays and weekends were collected through questionnaires, and the duration of different intensity physical activities was calculated using the Compendium of Physical Activities for Youth [33,34]. Activities with Metabolic Equivalents (METs) ≥ 3 were classified as moderate-to-vigorous physical activity (MVPA) [35]. The MVPA for weekdays and weekends was totaled and then divided by 7 to calculate the average daily MVPA. An average daily MVPA of ≥60 min was considered to meet the physical activity guideline [36].

The assessment of screen time encompassed common behaviors in daily life, including watching TV, using smartphones, playing video games, using computers, and browsing the internet. The total screen time for weekdays and weekends was summed and then divided by 7 to calculate the average daily screen time. An average daily screen time of ≤2 h was considered to meet the screen time guideline [36].

In the CHNS, participants’ sleep duration was assessed with the following question: “How many hours do you usually sleep each day, including both night and daytime sleep?” For participants under 13 years old, an average sleep duration of 9–11 h, and for those aged 14–17 years, an average sleep duration of 8–10 h was considered to meet the sleep guideline [36].

### 2.4. Assessment of Covariates

The covariates include age, sex, body mass index (BMI), region (north/south China), place of residence (urban/rural), and household net income category (low/high). Trained study workers measured participants’ height (cm) and weight (kg), and BMI was calculated using the formula: BMI = weight (kg)/height (m)^2^. The household net income categories are based on the median household net income. Participants are classified as high if their household net income exceeds the median; otherwise, they are classified as low.

### 2.5. Statistical Analysis

Continuous variables were summarized as mean ± standard deviation, and differences were tested using independent samples *t*-tests. Categorical variables were expressed as frequency (percentage, %) and compared using chi-square tests. Adjusted logistic regression models, controlling for covariates, were used to analyze the association between adherence to the 24-h movement behavior guidelines (meeting vs. not meeting) and the adequacy of macronutrient intake relative to DRIs (categorized as below, meeting, or above the reference). Additionally, covariate-adjusted restricted cubic spline (RCS) regression was employed to explore potential dose–response relationships between 24-h movement behaviors and: (1) macronutrient DRI adequacy (below, meeting, above), (2) macronutrient intakes, and (3) E% from each macronutrient. The RCS models used four knots at the 5th, 35th, 65th, and 95th percentiles. When analyzing the relationship between a specific 24-h movement behavior (e.g., PA) and macronutrient variables, adjustments included common sociodemographic factors as well as the other two movement behaviors (e.g., ST and SLP). All statistical analyses were performed using SPSS version 24.0 (IBM, Armonk, NY, USA) and R software version 4.4.2 (R Foundation for Statistical Computing, Vienna, Austria). A two-sided *p*-value < 0.05 was considered statistically significant.

## 3. Results

### 3.1. Social-Demographic Characteristics

Among the 3624 participants included in the analysis, the mean age was 10.9 years, and 49.9% were male. Detailed characteristics are presented in Table 1.

### 3.2. Adherence to 24-h Movement Guidelines Components and Macronutrient Intake

Table 2 shows the macronutrient intake profiles of participants grouped by their adherence to PA, ST, and SLP guidelines. The results indicate that participants who meet the PA guideline have significantly higher daily energy intake, carbohydrate intake (both in total and E%), and fat intake (both in total and E%) compared to those who do not meet the PA guideline (*p* < 0.001). On the other hand, E% from protein is significantly lower in participants meeting the PA guideline (*p* < 0.001). Participants meeting the ST guideline showed significantly higher carbohydrate intake and E% from carbohydrates (*p* < 0.05), while demonstrating a lower E% from fat (*p* < 0.001) compared to those not meeting the ST guideline. Additionally, participants who adhere to the SLP guideline have significantly lower protein intake and E% from protein compared to those who do not meet this guideline (*p* < 0.05). 

### 3.3. Associations Between Adherence to 24-h Movement Guidelines Components and Macronutrient DRIs Compliance

Table 3 presents the associations between adherence to 24-h movement guidelines components and carbohydrate DRIs. Multivariable logistic regression analysis found that participants who met the PA guideline were significantly less likely to consume carbohydrates above the DRIs compared to those who did not meet the PA guideline (odds ratio (OR) = 0.83, 95% confidence interval (CI): 0.69–0.99). In contrast, participants who met the ST guideline were significantly more likely to consume carbohydrates above the DRIs than those who did not meet the ST guideline (OR = 1.32, 95% CI: 1.11–1.56). Table 4 illustrates the associations between adherence to the 24-h movement guidelines components and fat DRIs. Multivariable logistic regression analysis showed that participants meeting the PA guideline had a significantly higher likelihood of fat intake above the DRIs compared to those not meeting the guideline (OR = 1.20, 95% CI: 1.02–1.40). Conversely, participants who met the ST guideline had a significantly lower likelihood of fat intake above DRIs compared to those who did not meet the guideline (OR = 0.78, 95% CI: 0.68–0.91). As shown in Table 5, no significant associations were observed between adherence to any of the 24-h movement guidelines components and protein DRIs. Additionally, no significant relationships were found between the number of guidelines met and macronutrient intake adequacy relative to DRIs (Tables S1–S3).

### 3.4. Dose–Response Associations Between 24-h Movement Behaviors and Macronutrient DRIs

**Figure 2:**
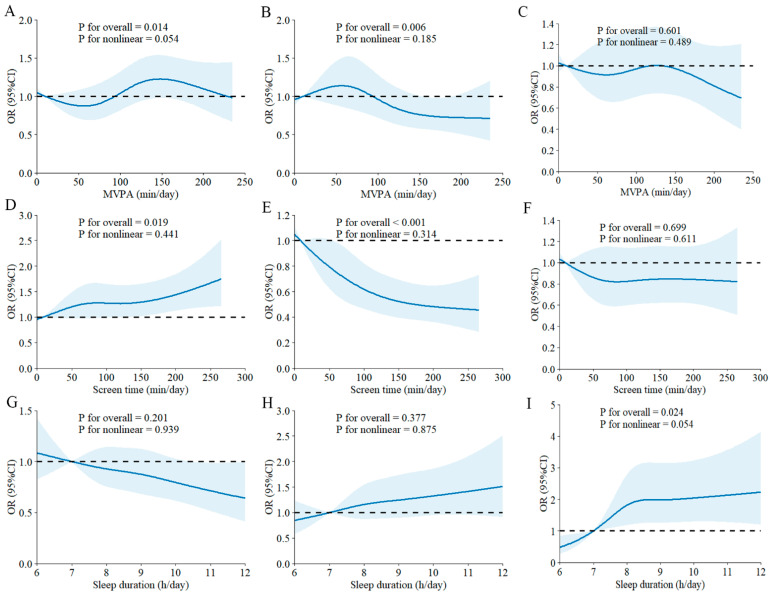
Dose–response associations between 24-h movement behaviors and macronutrient intake below DRIs. (**A**–**C**) MVPA with carbohydrate, fat, and protein intakes below DRIs; (**D**–**F**) ST with carbohydrate, fat, and protein intakes below DRIs; (**G**–**I**) SLP with carbohydrate, fat, and protein intakes below DRIs. The blue line represents the fitted line and the dashed line represents the reference line. Abbreviations: MVPA, moderate-to-vigorous physical activity; DRIs, Dietary Reference Intakes; OR, odds ratio; CI, confidence interval.

**Figure 3:**
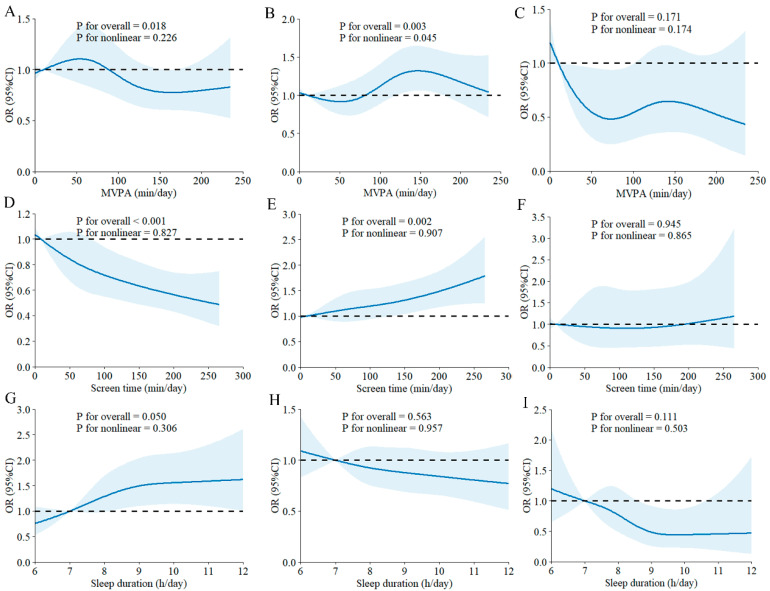
Dose–response associations between 24-h movement behaviors and macronutrient intake above DRIs. (**A**–**C**) MVPA with carbohydrate, fat, and protein intakes above DRIs; (**D**–**F**) ST with carbohydrate, fat, and protein intakes above DRIs; (**G**–**I**) SLP with carbohydrate, fat, and protein intakes above DRIs. The blue line represents the fitted line and the dashed line represents the reference line. Abbreviations: MVPA, moderate-to-vigorous physical activity; DRIs, Dietary Reference Intakes; OR, odds ratio; CI, confidence interval.

RCS analysis showed positive linear dose–response relationships between MVPA and ST, with carbohydrate intake below DRIs (Figure 2A,D). In contrast, negative linear dose–response associations were found between MVPA and ST, with fat intake below DRIs (Figure 2B,E). Additionally, SLP exhibited a positive dose–response relationship with protein intake below DRIs (Figure 2I). As shown in Figure 3A, MVPA had a negative linear dose–response relationship with carbohydrate intake above DRIs. However, a nonlinear dose–response association was observed between MVPA and fat intake above DRIs, with the risk of fat intake exceeding DRIs increasing with higher MVPA beyond approximately 90 min per day (Figure 3B). Furthermore, ST demonstrated a negative linear dose–response association with carbohydrate intake above DRIs (Figure 3D), while it showed a positive linear dose–response association with fat intake above DRIs (Figure 3E). Nonetheless, no significant dose–response relationships were found between any 24-h movement guidelines components and macronutrient intake meeting DRIs (Figure S1).

### 3.5. Dose–Response Associations Between 24-h Movement Behaviors and Macronutrient Intake, E% from Macronutrients

MVPA showed a negative linear dose–response association with E% from carbohydrates (Figure S2B), while positive linear dose–response relationships were observed between MVPA and both fat intake and E% from fats (Figure S2C,D). Figure S3 illustrates the dose–response relationships between ST and macronutrient intake as well as E% from macronutrients. ST exhibited negative linear dose–response relationships with both carbohydrate intake and E% from carbohydrates (Figure S3A,B). Conversely, ST displayed positive linear dose–response associations with both fat intake and E% from fats (Figure S3C,D). A U-shaped dose–response relationship was also observed between ST and protein intake (Figure S3E). Figure S4 illustrates the dose–response relationships between SLP and macronutrient intake as well as E% from macronutrients. SLP showed positive linear dose–response relationships with both carbohydrate intake and E% from carbohydrates (Figure S4A,B). An L-shaped dose–response relationship was found between SLP and E% from protein (Figure S4F).

## 4. Discussions

Based on population-representative data from the CHNS, the present study offers valuable insights into the relationships between 24-h movement behaviors and macronutrient intake among elementary and middle school students. This study revealed significant differences in energy and macronutrient intake among participants with different adherence patterns to the 24-h movement guidelines. Specifically, participants who met the PA guideline displayed higher dietary energy intake, along with increased intake of carbohydrate, fat, and protein, compared to those who did not meet the PA guideline. Participants meeting the ST guideline showed higher carbohydrate intake and a higher E% from carbohydrates, while having a lower E% from fat, compared to those not meeting the ST guideline. Additionally, participants who met the SLP guideline had lower protein intake and a lower E% from protein compared to those who did not meet the SLP guideline. Consistent with the current findings, Vilhar et al. [37] reported that energy and macronutrient intakes were higher in vigorously physically active adolescents than in those who were moderately or lightly active. Similarly, Dunton et al. [38] found that sedentary behavior was positively associated with the percentage of dietary fat intake and negatively associated with the percentage of dietary carbohydrate intake.

Previous studies have mainly analyzed 24-h movement behaviors and dietary intake as separate independent variables, while overlooking the potential relationship between them [9,14,20,39]. In this study, we compared macronutrient intake among participants with varying adherence to the components of 24-h movement guidelines. We further analyzed the associations between adherence to these guidelines and macronutrient DRIs compliance status (below, meet, above). The results showed that, compared to participants not meeting the PA guideline, those meeting the PA guideline were associated with a lower probability of carbohydrate intake above DRIs (OR = 0.83, 95% CI: 0.69–0.99), while also being associated with a higher likelihood of fat intake above DRIs (OR = 1.20, 95% CI: 1.02–1.40). One possible explanation for the higher probability of fat intake exceeding the DRIs among participants who meet the physical activity guidelines could be the increased total energy demands associated with higher levels of physical activity, which may subtly encourage a greater consumption of fats [37]. Additionally, the food reward mechanism may also be a possible explanation. Research has shown that even a single bout of exercise can lead to enhanced appetite sensation and food cravings, resulting in increased consumption of high-fat foods [40]. Although these participants showed higher fat intake, they often followed healthier dietary patterns, such as higher consumption of quality fats from dairy products. The increased fat intake may reflect one aspect of their overall healthy diet [41,42]. Additionally, we found that, compared to participants not meeting the ST guideline, those meeting the ST guideline had a higher probability of carbohydrate intake above the DRIs, while being less likely to have fat intake above the DRIs (OR = 0.78, 95% CI: 0.68–0.91) and more likely to have fat intake below the DRIs (OR = 1.40, 95% CI: 1.15–1.70). Consistent with our findings, previous research suggests that increased screen time is associated with a higher consumption of high-fat foods among children and adolescents. A study of Greek school-age children found that increased screen time was significantly associated with higher fast-food consumption and was also related to lower adherence to the Mediterranean dietary pattern and less frequent fruit intake [43]. Furthermore, recreational ST has been found to be associated with increased daily snacking, sugar-sweetened beverage consumption, and fast food intake [44], suggesting that “mindless eating” behaviors during screen use may contribute to excessive consumption of high-fat foods. Conversely, one study demonstrated that children and adolescents with less screen time had higher carbohydrate intake from fruits, vegetables, and whole grains [45], aligning with the findings of this study. Additionally, reduced screen time may be associated with more regular eating habits, thereby increasing opportunities for carbohydrate intake from vegetables and fruits, while decreasing the consumption of high-fat, high-calorie foods [46].

To date, studies investigating the associations between 24-h movement behaviors and dietary outcomes among children and adolescents are relatively limited [47]. Although research aimed at exploring dose–response relationships has increased in recent years [48,49,50,51], these studies did not take into account 24-h movement behaviors and dietary intake simultaneously. Very few studies have explored the dose–response relationships between 24-h movement behaviors and macronutrient intake, particularly in children and adolescents. In this study, we used a population-representative sample from CHNS to analyze the dose–response relationships between various 24-h movement behaviors and macronutrient intake. We found a nonlinear dose–response relationship between MVPA and fat intake above DRIs, where MVPA exceeding 90 min/day was associated with increased risk of fat intake above DRIs. Additionally, MVPA showed positive linear dose–response associations with both fat intake and energy percentage from fats, while demonstrating a negative dose–response association with E% from carbohydrates. Although research evidence suggests that appropriate increases in fat intake may benefit muscle mass and strength [52], and higher physical activity levels may prevent excessive fat accumulation despite increased fat consumption, physically active children and adolescents should focus on the types of dietary fats consumed, prioritizing high-quality fat sources. Furthermore, we found a negative linear dose–response association between ST and carbohydrate intake above DRIs. In contrast, positive linear dose–response associations were observed between ST and fat intake above DRIs, fat intake, and E% from fats. This suggests that children and adolescents with excessive screen use should consider limiting fat intake while increasing carbohydrate intake in their daily diet to avoid macronutrient imbalance caused by excessive fat consumption. Consistent with our study, Falbe et al. [53] found that each hour per day increase in total screen time among adolescents increased intake of high-fat, high-calorie foods. In another study of Japanese children and adolescents [54], increased screen time was associated with increased fat intake and decreased carbohydrate intake. Current research has not reached a consensus regarding the relationship between sleep duration and macronutrient intake. Some studies found that longer sleep duration was associated with lower carbohydrate intake and energy percentage from carbohydrates, but higher fat intake and energy percentage from fats [55,56]. However, another study indicated that longer sleep duration was associated with greater carbohydrate intake and decreased fat intake [57]. In the present study, SLP showed positive linear dose–response associations with both carbohydrate intake and energy percentage from carbohydrates. The inconsistent findings across studies may first be attributed to differences in sleep measurement methods (self-report versus objective measurement). Additionally, most previous studies did not adequately control for variables such as physical activity and screen time, which may explain the discrepancies between our results and some earlier studies. Moreover, we found that increased sleep duration was associated with protein intake below DRIs, and sleep duration demonstrated an approximately “L-shaped” dose–response relationship with energy percentage from protein. This phenomenon may primarily be related to decreased total energy intake associated with adequate sleep duration. Haszard et al. [58] found that a 48-min reduction in children’s sleep could increase total energy intake by 62.6 kcal. Conversely, a randomized controlled trial in adults found that the sleep extension group had significantly reduced total energy intake compared to the control group [59]. Furthermore, research indicates that sleep deprivation disrupts hormones regulating hunger (such as ghrelin and leptin), increasing hunger and appetite, thereby promoting intake of high-energy, high-protein foods [60]. Therefore, the declining trends in protein intake and E% from protein with increased sleep duration are expected.

To our knowledge, this study is the first to examine the association between adherence to the 24HMG components (MVPA, ST, and SLP) and macronutrient intake, as well as the dose–response associations between these movement behaviors and macronutrient intake among students aged 6–17 years. However, this study has several limitations. First, dietary data were collected through three consecutive 24-h dietary recalls, which cannot entirely avoid errors caused by individual dietary habits and recall bias. Second, although the CHNS included participants from 15 provinces in China, some provinces were still excluded, making this study not fully nationally representative. Third, this study is based on CHNS data from 2004–2011, which has a lag of more than 10 years from the present, and may not accurately reflect the current status of 24-h movement behaviors and macronutrient intake among Chinese children and adolescents. This is an important limitation of this study. However, the China Health and Nutrition Survey is the only database that meets the requirements of our study design—namely, having a sufficient sample of children and adolescents, the necessary variables, and strong population representativeness. Therefore, there are no newer databases on which the assumptions of this type of research could be based, which is why the study was based on data from 2004–2011. Additionally, even though we adjusted for some covariates in our analysis, there are still some factors that were not considered in this study due to lack of measurement, such as food quality or nutrient subtypes (e.g., saturated vs. unsaturated fat, plant vs. animal protein), PA accumulation patterns, and sleep quality, which may somewhat limit the interpretability of the results. Finally, since this study is cross-sectional, it cannot establish causal relationships between adherence to the 24HMG components and macronutrient intake. Future research should consider larger-scale national longitudinal surveys to explore causal relationships more thoroughly.

## 5. Conclusions

Among Chinese children and adolescents aged 6–17 years, those who meet the PA guideline need to be more concerned about the risk of excessive fat intake, whereas those who meet the ST guideline need to pay attention to the risk of excessive carbohydrate intake in their daily diet. With increasing ST, the risks of insufficient carbohydrate intake and excessive fat intake rise linearly. There was a nonlinear dose–response association between MVPA and excessive fat intake. To obtain health benefits through engaging in PA while preventing increased risk of excessive fat intake, MVPA should be maintained within 60–90 min/day. The findings of this study emphasize the importance of adjusting macronutrient proportions in the daily diet according to 24-h movement behavior guideline adherence to avoid macronutrient intake imbalance. Furthermore, this study determined the optimal MVPA range after comprehensively considering PA guideline and macronutrient intake factors, providing a theoretical reference for the development of future integrated guidelines for 24-h movement behaviors and dietary intake.

## Figures and Tables

**Table 1:** Basic characteristics of study participants.

Characteristics *n* = 3624	Mean ± SD/*n* (%)
Age (years)	10.9 ± 3.1
BMI (kg/m^2^)	17.6 ± 3.2
Sex	
Boys	1809 (49.9)
Girls	1815 (50.1)
Region	
North	1403 (38.7)
South	2221 (61.3)
Place of residence	
Urban	1079 (29.8)
Rural	2545 (70.2)
Family net income category	
Low	1811 (50.0)
High	1813 (50.0)

Abbreviations: BMI, body mass index.

**Table 2:** Macronutrient intake among participants with different 24-h movement guidelines components adherence.

	PA Guideline			ST Guideline			SLP Guideline		
	Meeting (*n* = 2551)	Not Meeting Guideline (*n* = 1073)	*p*	Meeting (*n* = 2479)	Not Meeting (*n* = 1145)	*p*	Meeting (*n* = 2702)	Not Meeting (*n* = 922)	*p*
Energy (kcal/day)	1723.0 ± 571.1	1567.8 ± 540.6	**<0.001**	1686.4 ± 579.4	1656.8 ± 537.4	0.133	1669.4 ± 563.6	1699.5 ± 574.7	0.163
Carbohydrate (g/day)	242.5 ± 91.7	227.3 ± 92.1	**<0.001**	241.0 ± 94.5	231.4 ± 86.1	**0.003**	237.7 ± 92.5	238.9 ± 90.9	0.737
Fat (g/day)	59.1 ± 31.2	50.8 ± 29.3	**<0.001**	56.1 ± 31.5	57.7 ± 29.3	0.172	56.2 ± 29.9	58.0 ± 33.4	0.153
Protein (g/day)	54.8 ± 21.1	50.0 ± 19.2	**<0.001**	53.8 ± 21.1	52.6 ± 19.7	0.105	52.8 ± 20.3	55.2 ± 21.7	**0.003**
Carbohydrate (E%)	56.6 ± 11.7	58.2 ± 12.0	**<0.001**	57.5 ± 12.0	56.1 ± 11.3	**0.001**	57.2 ± 11.7	56.7 ± 12.2	0.325
Fat (E%)	30.4 ± 11.2	28.7 ± 11.7	**<0.001**	29.5 ± 11.6	30.9 ± 10.8	**0.001**	29.9 ± 11.2	30.0 ± 11.8	0.778
Protein (E%)	12.8 ± 2.9	12.9 ± 3.0	**<0.001**	12.8 ± 3.0	12.8 ± 2.9	0.824	12.7 ± 2.9	13.1 ± 3.1	**0.005**

Abbreviations: PA, physical activity; ST, screen time; SLP, sleep; E%, percentage of dietary energy intake.

**Table 3:** Association between 24-h movement guidelines components adherence and carbohydrate DRIs.

	Carbohydrate Intake Below DRIs		Carbohydrate Intake Meeting DRIs		Carbohydrate Intake Above DRIs	
	OR (95% CI)	*p*	OR (95% CI)	*p*	OR (95% CI)	*p*
PA						
Not meeting the guideline	Reference		Reference		Reference	
Meeting the guideline	1.15 (0.98–1.35)	0.072	1.01 (0.86–1.19)	0.820	**0.83 (0.69–0.99)**	**0.039**
ST						
Not meeting the guideline	Reference		Reference		Reference	
Meeting the guideline	0.86 (0.74–1.00)	0.055	0.94 (0.81–1.09)	0.436	**1.32 (1.11–1.56)**	**0.002**
SLP						
Not meeting the guideline	Reference		Reference		Reference	
Meeting the guideline	1.03 (0.87–1.21)	0.693	0.90 (0.77–1.06)	0.237	1.09 (0.91–1.31)	0.324

Abbreviations: PA, physical activity; ST, screen time; SLP, sleep; DRIs, Dietary Reference Intakes; OR, odds ratio; CI, confidence interval.

**Table 4:** Association between 24-h movement guidelines components adherence and fat DRIs.

	Fat Intake Below DRIs		Fat Intake Meeting DRIs		Fat Intake Above DRIs	
	OR (95% CI)	*p*	OR (95% CI)	*p*	OR (95% CI)	*p*
PA						
Not meeting the guideline	Reference		Reference		Reference	
Meeting guideline	0.83 (0.69–1.01)	0.066	0.94 (0.80–1.10)	0.442	**1.20 (1.02–1.40)**	**0.020**
ST						
Not meeting the guideline	Reference		Reference		Reference	
Meeting the guideline	**1.40 (1.15–1.70)**	**0.001**	1.04 (0.89–1.12)	0.594	**0.78 (0.68–0.91)**	**0.001**
SLP						
Not meeting the guideline	Reference		Reference		Reference	
Meeting the guideline	1.01 (0.83–1.24)	0.872	0.95 (0.81–1.12)	0.602	1.03 (0.88–1.21)	0.656

Abbreviations: PA, physical activity; ST, screen time; SLP, sleep; DRIs, Dietary Reference Intakes; OR, odds ratio; CI, confidence interval.

**Table 5:** Association between 24-h movement guidelines components adherence and protein DRIs.

	Protein Intake Below DRIs		Protein Intake Meeting DRIs		Protein Intake Above DRIs	
	OR (95% CI)	*p*	OR (95% CI)	*p*	OR (95% CI)	*p*
PA						
Not meeting the guideline	Reference		Reference		Reference	
Meeting the guideline	1.00 (0.81–1.23)	0.986	1.05 (0.86–1.28)	0.577	0.73 (0.47–1.15)	0.186
ST						
Not meeting the guideline	Reference		Reference		Reference	
Meeting the guideline	1.01 (0.82–1.23)	0.919	1.01 (0.83–1.21)	0.920	0.91 (0.59–1.40)	0.671
SLP						
Not meeting the guideline	Reference		Reference		Reference	
Meeting the guideline	1.17 (0.93–1.46)	0.170	0.95 (0.78–1.17)	0.681	0.66 (0.43–1.02)	0.063

Abbreviations: PA, physical activity; ST, screen time; SLP, sleep; DRIs, Dietary Reference Intakes; OR, odds ratio; CI, confidence interval.

## Data Availability

The data were obtained from the CHNS. The original database is available at the website (https://www.cpc.unc.edu/projects/china) (accessed on 1 August 2025).

## Supplementary Materials

The following supporting information can be downloaded at: https://www.mdpi.com/article/10.3390/nu17203262/s1, Table S1: Association between the meeting number of 24-h movement guidelines components and carbohydrates DRIs, Table S2: Association between the meeting number of 24-h movement guidelines components and fats DRIs, Table S3: Association between the meeting number of 24-h movement guidelines components and proteins DRIs, Figure S1: Dose–response associations between 24-h movement behaviors and macronutrient intake meeting DRIs, Figure S2: Dose–response associations between MVPA and macronutrient intake and E% from macronutrient, Figure S3: Dose–response associations between ST and macronutrient intake and E% from macronutrient, Figure S4: Dose–response associations between SLP and macronutrient intake and E% from macronutrient.
