# Supplementary material for: Associations Between 24-h Movement Behaviors and Macronutrient Intake Among Students Aged 6–17 Years: Insights from the China Health and Nutrition Survey
Source: Nutrients. 2025 Oct 17;17(20):3262. doi: 10.3390/nu17203262 (PMC12567218; doi:10.3390/nu17203262)
Supplement: Supplementary file 1 [file nutrients-17-03262-s001.zip › nutrients-3917508-supplementary.pdf]

Supplementary Materials

Table S1. Association between the number of meetings of the 24-hour movement guidelines components and carbohydrate DRIs.

	Carbohydrate intake below DRIs		Carbohydrate intake meeting DRIs		Carbohydrate intake above DRIs	
	**OR (95% CI)**	***p***	**OR (95% CI)**	***p***	**OR (95% CI)**	***p***
Meeting one	Reference		Reference		Reference	
Meeting two	1.01 (0.83 − 1.23)	0.892	0.95 (0.78 − 1.15)	0.605	1.05 (0.84 − 1.31)	0.657
Meeting three	1.00 (0.82 − 1.22)	0.971	0.91 (0.74 − 1.12)	0.384	1.13 (0.90 − 1.42)	0.288

Abbreviations: DRIs, Dietary Reference Intakes; OR, odds ratio; CI, confidence interval.

Table S2. Association between meeting the 24-hour movement guidelines components and fat DRIs.

	Fat intake below DRIs		Fat intake meeting DRIs		Fat intake above DRIs	
	**OR (95% CI)**	***p***	**OR (95% CI)**	***p***	**OR (95% CI)**	***p***
Meeting one	Reference		Reference		Reference	
Meeting two	1.19 (0.93 − 1.53)	0.151	0.90 (0.74 − 1.09)	0.288	0.99 (0.82 − 1.19)	0.926
Meeting three	1.16 (0.90 − 1.50)	0.247	0.93 (0.76 − 1.11)	0.506	0.98 (0.80 − 1.19)	0.856

Abbreviations: DRIs, Dietary Reference Intakes; OR, odds ratio; CI, confidence interval.

Table S3. Association between the number of meetings for 24-hour movement guidelines components and protein DRIs.

	Protein intake below DRIs		Protein intake meeting DRIs		Protein intake above DRIs	
	**OR (95% CI)**	***p***	**OR (95% CI)**	***p***	**OR (95% CI)**	***p***
Meeting one	Reference		Reference		Reference	
Meeting two	0.84 (0.64 − 1.09)	0.199	1.18 (0.92 − 1.51)	0172	0.88 (0.52 − 1.51)	0.662
Meeting three	1.10 (0.84 − 1.44)	0.462	0.99 (0.77 − 1.27)	0.947	0.65 (0.36 − 1.16)	0.152

Abbreviations: DRIs, Dietary Reference Intakes; OR, odds ratio; CI, confidence interval.

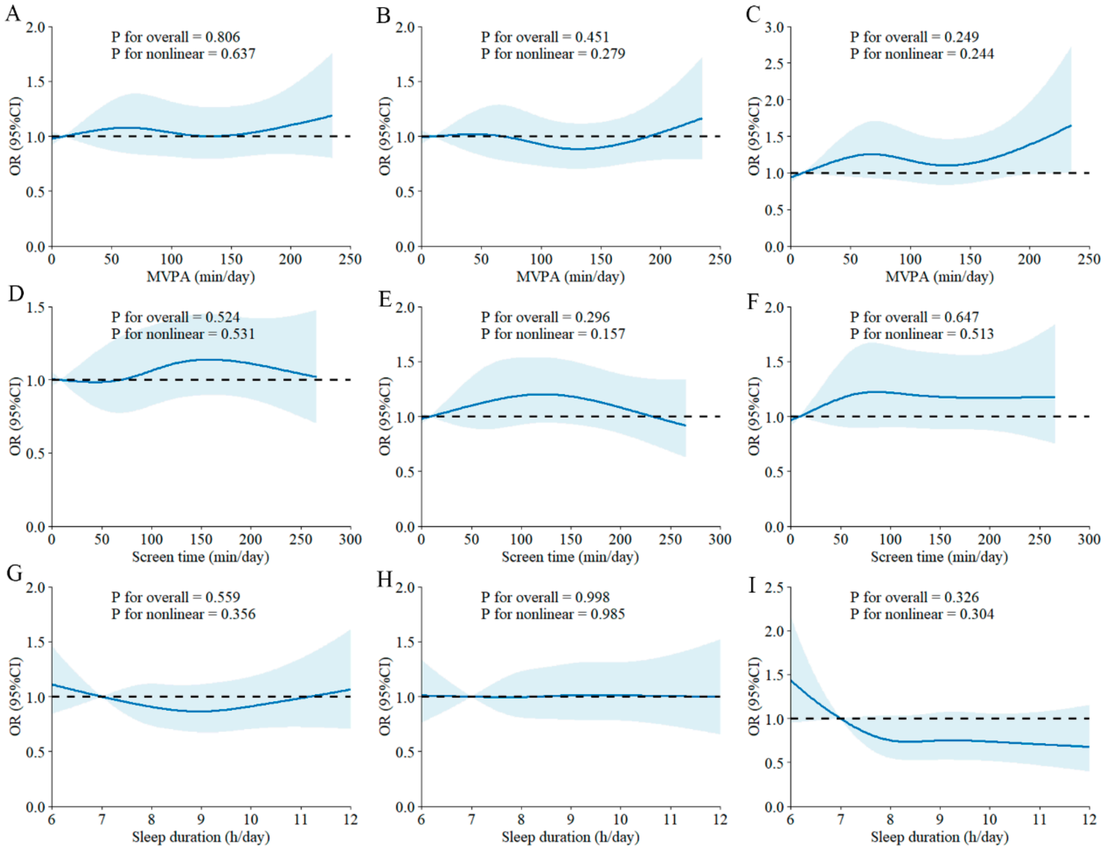

Figure S1. Dose-response associations between 24-hour movement behaviors and macronutrient intake meeting DRIs.（A-C）MVPA with carbohydrates, fats, and proteins intake meet DRIs; (D-F) ST with carbohydrates, fats, and proteins intake meet DRIs; (G-I) SLP with carbohydrates, fats, and proteins intake meet DRIs. Abbreviations: MVPA, moderate-to-vigorous physical activity; DRIs, Dietary Reference Intakes; OR, odds ratio; CI, confidence interval.

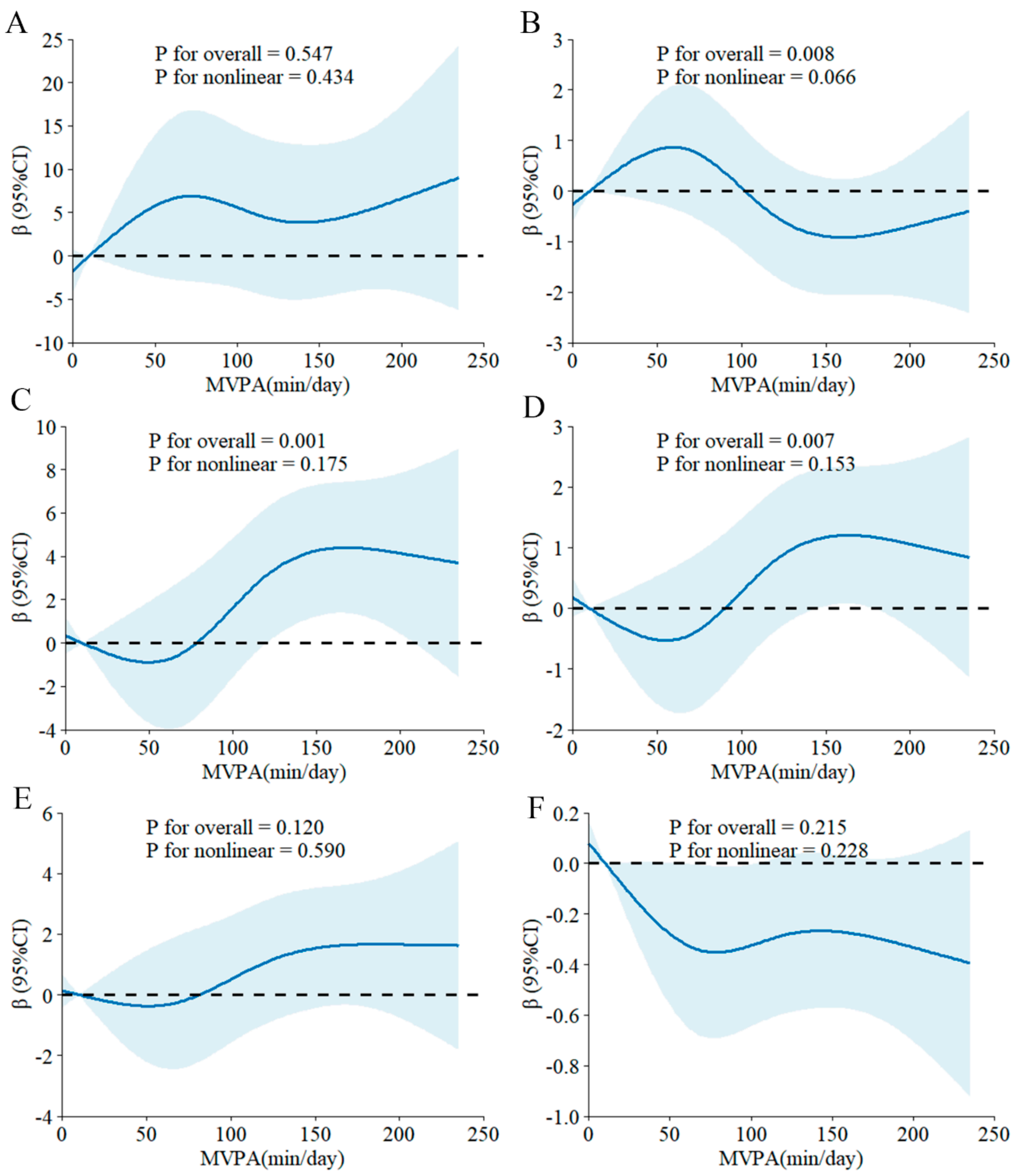

Figure S2. Dose-response associations between MVPA and macronutrient intake and E% from macronutrient. (A-B) MVPA with carbohydrate intake, E% from carbohydrates; (C-D) MVPA with fat intake, E% from fats; (E-F) MVPA with protein intake, E% from proteins. Abbreviations: MVPA, moderate-to-vigorous physical activity; E%, percentage of dietary energy intake; OR, odds ratio; CI, confidence interval.

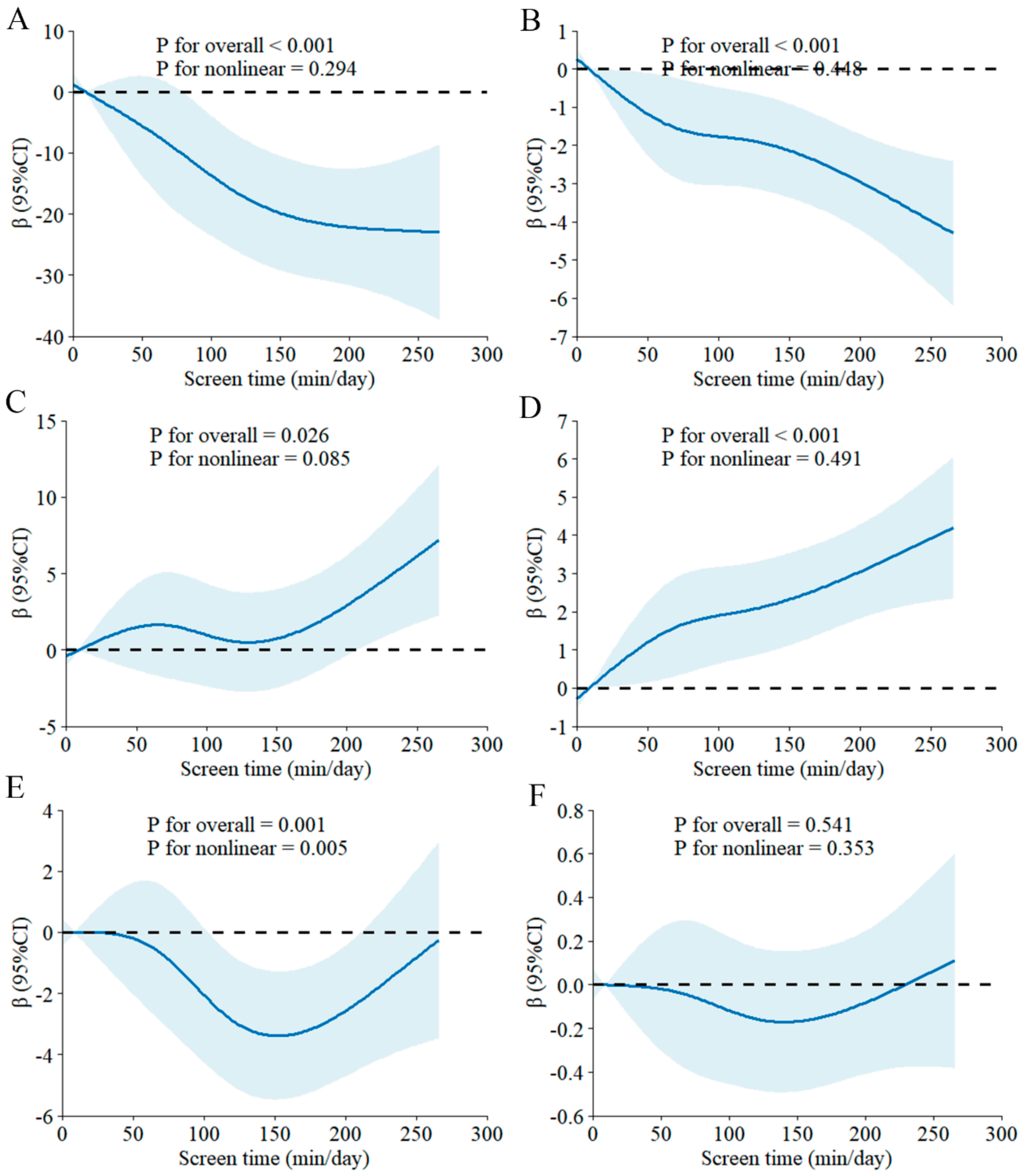

Figure S3. Dose-response associations between ST and macronutrient intake and E% from macronutrient. (A-B) ST with carbohydrate intake, E% from carbohydrates; (C-D) ST with fat intake, E% from fats; (E-F) ST with protein intake, E% from proteins. Abbreviations: MVPA, moderate-to-vigorous physical activity; E%, percentage of dietary energy intake.

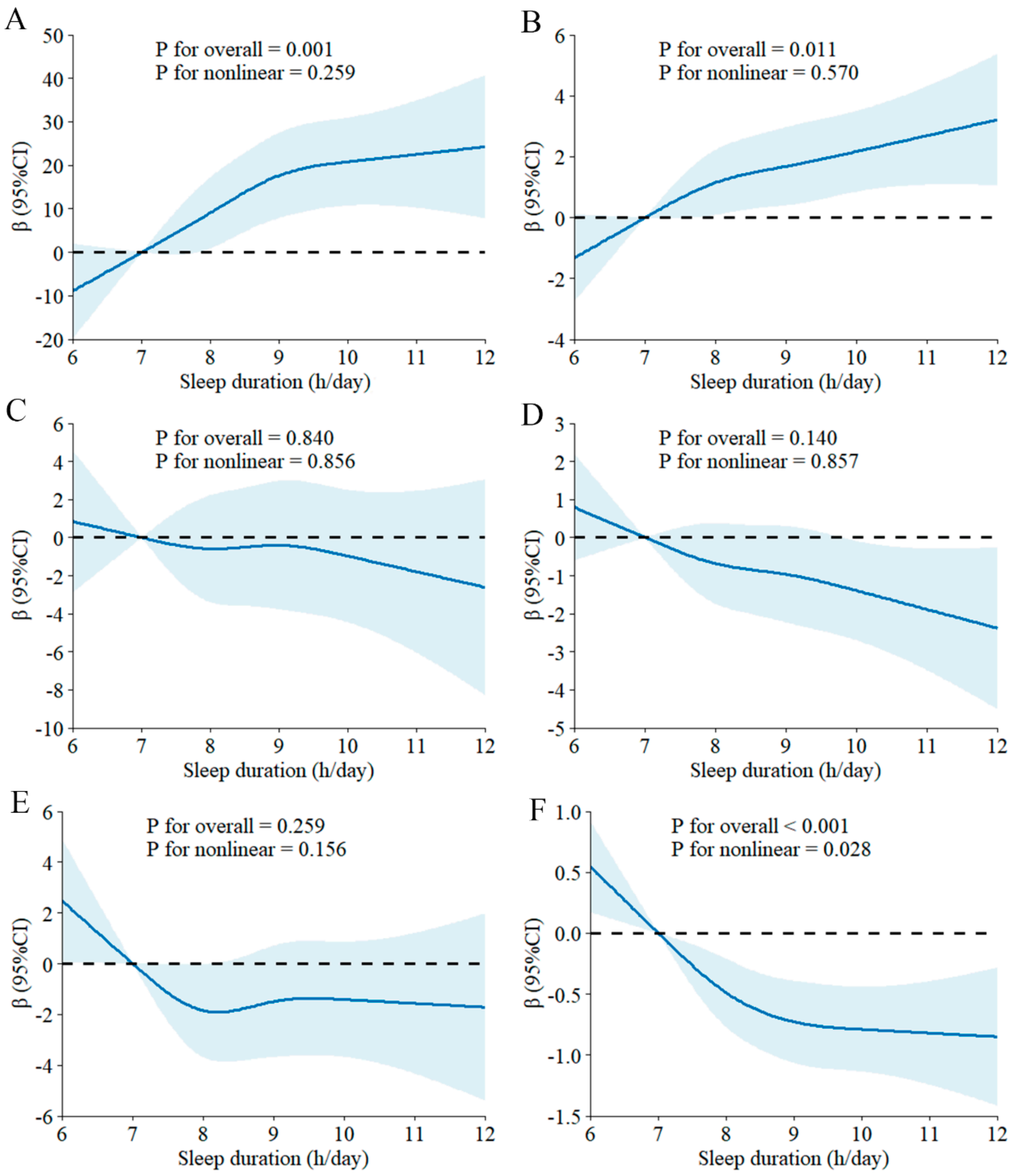

Figure S4. Dose-response associations between SLP and macronutrient intake and E% from macronutrient. (A-B) SLP with carbohydrate intake, E% from carbohydrates; (C-D) SLP with fat intake, E% from fats; (E-F) SLP with protein intake, E% from proteins. Abbreviations: MVPA, moderate-to-vigorous physical activity; E%, percentage of dietary energy intake.
